# Dietary Patterns and Dietary Adaptations in Women With and Without Gestational Diabetes: Evidence From the Growing Up in New Zealand Study

**DOI:** 10.3390/nu12010227

**Published:** 2020-01-15

**Authors:** Robyn L. Lawrence, Clare R. Wall, Frank H. Bloomfield

**Affiliations:** 1 The Liggins Institute, University of Auckland, Private Bag 92019, Auckland 1142, New Zealand; robyn.lawrence@auckland.ac.nz; 2 Discipline of Nutrition and Dietetics, School of Medical Sciences, University of Auckland, Private Bag 92019, Auckland 1142, New Zealand; c.wall@auckland.ac.nz

**Keywords:** gestational diabetes, dietary patterns, pregnancy, maternal nutrition, dietary adaptations

## Abstract

Diet is a cornerstone of the management of gestational diabetes (GDM). We investigated differences in dietary patterns and dietary adaptations among pregnant women with and without GDM participating in the Growing Up in New Zealand study. Presence of GDM was determined using coded clinical data and plasma glucose results meeting the New Zealand Society for the Study of Diabetes diagnostic criteria. Women answered a food frequency questionnaire and questions regarding dietary changes and information received during pregnancy. Women with GDM had lower adherence scores than those without GDM for ‘Junk’ (mean (SD) score −0.28 (0.95) versus 0.02 (1.01) *p* < 0.0005) and ‘Traditional/White bread’ dietary patterns (−0.18 (0.93) versus 0.01 (1.01) *p* = 0.002). More women with GDM reported avoiding foods high in fat or sugar (25.3% versus 5.7%, *p* < 0.05) compared to women without GDM. A greater proportion of women with GDM compared with those without GDM received information from dietitians or nutritionists (27.0% versus 1.7%, *p* < 0.05) or obstetricians (12.6% versus 7.5%, *p* < 0.05). More women diagnosed before the antenatal interview received advice from dietitians or nutritionists compared with those diagnosed after (46.9% versus 6.0%, *p* < 0.05). Women with GDM appear to make positive changes to their diet in response to advice received from health care professionals.

## 1. Introduction

Pregnancy is a time when women frequently pay extra attention to their diet in order to promote the health and well-being of themselves and their baby [1,2,3,4]. Women receive information from a range of sources [2,3] and make a number of dietary adaptations during pregnancy [5]. Diet is thought to play a critical role in the development of gestational diabetes mellitus (GDM), a form of carbohydrate intolerance first diagnosed in pregnancy [6], and a number of dietary components have been associated with an increased or decreased risk of GDM [7,8,9,10,11]. GDM poses significant health risks to both mother and infant [12,13,14] extending beyond the pregnancy and neonatal period [15,16]. Rates of GDM-affected pregnancies are increasing, thought to be at least in part due to concomitant increasing prevalence of overweight and obesity [17,18,19]. Globally, reported prevalence of GDM ranges between 1% and 45% of pregnancies [20,21,22]. In New Zealand, GDM has been estimated to affect approximately 6% of pregnancies [23].

Dietary pattern analysis has become a popular tool for exploring dietary associations with GDM, as it is thought to better reflect real eating behaviours by considering the eating pattern as a whole [24]. A number of studies have found dietary patterns characterised by high intakes of red and processed meats, fried foods and added sugars to be associated with an increased risk of GDM, while dietary patterns characterised by high consumption of fruit, vegetables, wholegrains and nuts or ‘Mediterranean’-style dietary patterns to be associated with a lower risk of developing GDM [25,26,27,28,29,30,31,32,33,34,35]. Nonetheless, research of dietary patterns and risk of GDM has primarily been conducted in largely Caucasian populations [11,25,30,34,36], with many using data from the same cohort of women from the Nurses’ Health Study II [25,28,36]. Ethnicity is a widely accepted risk factor for GDM [37], with women of non-European descent disproportionately affected [38]. Different ethnic populations tend to have different diets [39], which may further influence the risk of developing GDM. Dietary patterns and adherence to nutrition recommendations have been reported to differ amongst pregnant women of different ethnicities within New Zealand [40,41]; however, associations between diet and the development of GDM have not been explored in the New Zealand population. Whether women who develop GDM make similar dietary adaptations during pregnancy to those who do not develop GDM is also unknown. The aim of this study was therefore to explore differences in dietary patterns and dietary adaptations among women with and without a diagnosis of GDM during pregnancy in New Zealand.

## 2. Materials and Methods

### 2.1. Study Population

Data used for the analyses in this study were derived from 6822 women enrolled in the Growing Up in New Zealand study (www.growingup.co.nz), a pre-birth, longitudinal cohort study exploring multidisciplinary determinants of health and development for children born in New Zealand [42]. Pregnant women with an estimated due date between 25th April 2009 and 25th March 2010 residing in an area defined by the geographical boundaries of three regional health boards in the upper-mid North Island of New Zealand were eligible to participate in the study. The geographical area of recruitment was chosen for its ethnic, socioeconomic and urban and rural residency diversity with the aim of having a study cohort that was broadly generalizable to the rest of New Zealand [42]. Ethical approval was provided by the Ministry of Health Northern Y Regional Ethics Committee (reference NTY/08/06/055) and written informed consent was obtained from all participating women.

### 2.2. Data Collection

Data collection during the antenatal period comprised a face-to-face interview, collecting information on maternal demographics, health and pregnancy history, smoking status, dietary intake and physical activity. A total of 6822 women consented and completed the antenatal interview (most often during the third trimester of pregnancy) and 6657 consented to access to their routine health records through use of their unique National Hospital Identifier (NHI).

### 2.3. Measurements

#### 2.3.1. Diabetes Status during Pregnancy

The methods used to identify women with GDM in the cohort have been described previously [23]. Briefly, participant NHIs were used to obtain data on diabetes status during pregnancy from the Ministry of Health’s National Minimum Dataset, the three regional health boards, and laboratories servicing the recruitment catchment area. Coded clinical data were collected from the Ministry of Health and the three regional health boards. Laboratories provided fasting plasma glucose concentration, glucose challenge test results and glucose tolerance test results. Women were classified as having GDM if they had a clinical code for GDM or if they had a blood glucose result (between 12 weeks’ gestation to the end of pregnancy) meeting the diagnostic criteria for GDM in use by their regional health board at the time. All three regional health boards used the New Zealand Society for the Study of Diabetes criteria [43,44] to diagnose GDM: fasting plasma glucose of ≥ 5.5 mmol/L or a 2 h plasma glucose ≥ 9.0 mmol/L post 75 g oral glucose tolerance test (OGTT) [45,46,47,48]. One regional health board also considered a 60 min plasma glucose result on the 50 g glucose challenge test (GCT) of ≥ 11.1 mmol/L to be indicative of GDM [45]. Women identified as having pre-existing diabetes or impaired glucose tolerance were excluded from analyses.

#### 2.3.2. Dietary Patterns and Dietary Habits

A semi-quantitative 44 item food frequency questionnaire (FFQ) administered as part of the antenatal interview was used to collect information on dietary intake and has been described in detail elsewhere [40,41]. The purpose of the antenatal FFQ was to describe the frequency of consumption over the previous four weeks of the four core food groups as recommended by the New Zealand Ministry of Health’s guidelines for pregnant women [49]: fruits and vegetables; breads and cereals; milk, milk products, lean meat, meat alternatives and eggs, and foods likely to be high in fats, sugars and/or salt. Four dietary patterns have previously been identified in the cohort using principal component analysis as described by Wall et al. [41]. The dietary patterns identified were labelled as ‘Junk’, ‘Health conscious’, ‘Traditional/White bread’ and ‘Fusion/Protein’. Food items with factor loadings of 0.3 or greater in the principal component analysis were considered to be strongly associated with the identified pattern. The ‘Junk’ dietary pattern had high loadings of confectionary, snacks, takeaways, hot chips, processed meats, soft and energy drinks, battered fried fish or seafood, ice-cream and cakes or biscuits. The ‘Health conscious’ dietary pattern had high loadings of vegetables, cheese, brown wholemeal bread, non-citrus fruits, yoghurt, dried fruits, high fibre cereal, and Vegemite™ or Marmite™. The ‘Traditional/White bread’ had high factor loadings for whole or standard milk, white bread, margarine, jam honey marmalade, peanut butter, Nutella™ and low fibre and/or high sugar cereals. The ‘Fusion/Protein’ had high factor loadings for noodles, rice, pasta, seafood, chicken, green leafy vegetables, eggs and red meat. Summary scores for each dietary pattern were available for 5664 women who had antenatal dietary data. A higher score indicates a stronger adherence to that dietary pattern.

The antenatal questionnaire also included open questions regarding foods or drinks deliberately avoided or added to the diet due to pregnancy [50] and were categorised as ‘breads and cereals’, ‘lean meat, chicken, seafood, eggs, cooked dried beans, peas’, ‘milk and milk products’, ‘fruit and vegetables’, ‘supplement’, ‘chocolate’, ‘foods high in fat or sugar’, ‘alcohol’, ‘soft drinks’ and ‘other’. Women were asked whether they had received any information or been told anything that led them to make dietary changes while pregnant. If they answered ‘yes’ to this question they were asked to select from a list of information sources including ‘family/whānau’, ‘friends’, ‘GP (Family doctor)’, ‘midwife’, ‘obstetrician’, ‘dietitian/nutritionist’, alternative health practitioner’, ‘antenatal class’, ‘the internet’, ‘radio’, ‘TV’, ‘books, magazines, newspaper’, or ‘other’.

#### 2.3.3. Covariates

Questions relating to maternal socio-demographic, health and lifestyle characteristics were also included in the antenatal interview. Self-reported ethnicity was allocated to one of six Level 1 categories (i) European; (ii) Māori; (iii) Pacific Peoples; (iv) Asian; (v) Middle Eastern/Latin American/African (MELAA), and (vi) Other ethnicity according to the coding criteria used by Statistics New Zealand [51]. If women identified with multiple ethnicities but did not self-prioritise a primary ethnicity, the prioritisation methodology employed by Statistics New Zealand between 1991 to 2004 [52] was used, as mutually exclusive ethnic groups were required for statistical analyses. The ‘MELAA’ and ‘Other’ ethnic groups were combined into the ‘Other’ ethnic group due to small numbers in these groups. The New Zealand Deprivation Index (NZDep06) [53] was used as a measure of social deprivation. The index is divided into deciles from 1 (least deprived) to 10 (most deprived). Pre-pregnancy weight and height were self-reported and used to calculate pre-pregnancy body mass index (BMI). Weight gain during pregnancy up to the point of the antenatal interview was assessed in a question asking about weight change during pregnancy in 5 kg increments. Women were also asked whether they were actively dieting or trying to lose weight during the 6 months prior to pregnancy and whether or not they lost any weight during that time. Physical activity was assessed using questions from the International Physical Activity Questionnaire (IPAQ). Participants were asked about intensity (moderate or vigorous), duration (<30, 30 to 60, or >60 min) and frequency (days per week) of activity [54]. To be classified as participating in moderate or vigorous activity women had to have engaged in moderate activity for at least 30 min for at least five days per week or vigorous activity for at least 30 min on at least two days per week. Women were asked whether they received any treatment to assist them in becoming pregnant and, if women answered ‘yes’, this question was followed by a multiple response question relating the type of treatment given, which included ‘fertility awareness and weight loss’.

### 2.4. Statistical Analyses

Maternal socio-demographic, health and lifestyle characteristics and dietary patterns are reported as the frequency (%) for categorical variables and the mean ± standard deviation (SD) for continuous variables. In accordance with Growing Up in New Zealand data policy, cells where *n* < 10 are reported as <10 rather than the actual number. Differences in maternal characteristics and dietary patterns were tested using Chi squared or Fisher’s exact test and unadjusted and adjusted logistic regression for categorical variables and independent samples t-test for continuous variables. Results are reported as the mean (SD), frequency (%) or odds ratios (OR) or adjusted odds ratios (aOR) and 95% confidence intervals (CI). Maternal age (<35 and ≥35 years), ethnicity (European, Māori, Pacific, Asian, Other) NZDep06 score (1–3, 4–7 and 8–10), pre-pregnancy BMI (<25, 25–29.9 and ≥30 kg/m^2^), pre-pregnancy and first trimester physical activity (at least 150 min per week of moderate to vigorous physical activity), smoking pattern (continued smoking during pregnancy, stopped smoking during pregnancy, non-smoker), alcohol consumption (continued drinking during pregnancy, stopped drinking during pregnancy, non-drinker) and dietary pattern score were included in adjusted models. These variables were selected as they either were associated with GDM in univariate or multivariate analyses or are commonly considered to be associated with the risk of developing GDM in the literature. Analyses were conducted using SPSS version 21. A two-sided *p* value of <0.05 was considered statistically significant.

## 3. Results

**Figure 1:**
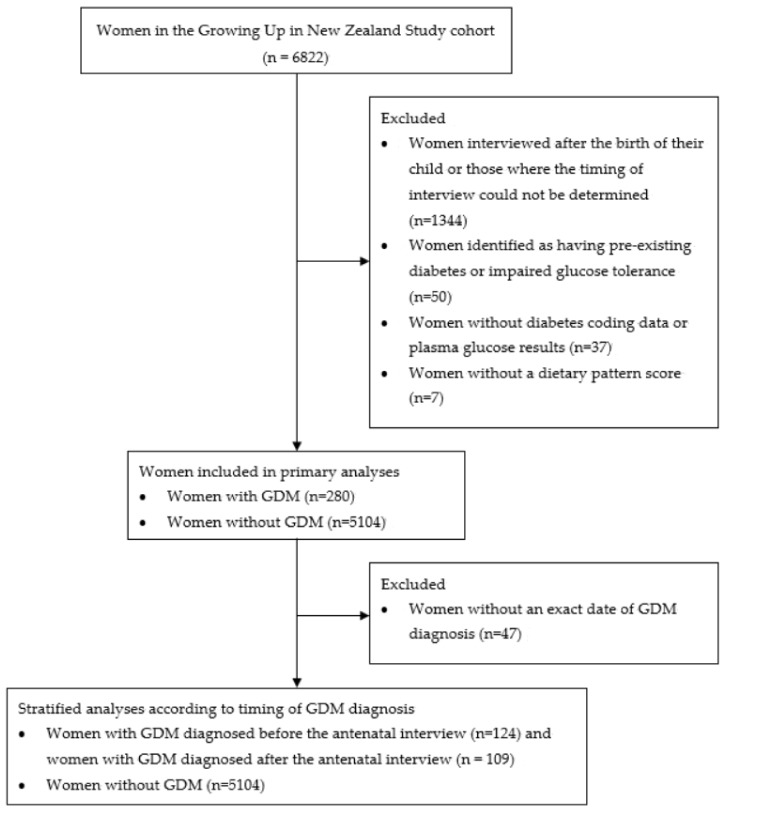
Flowchart showing selection of participants included in primary and secondary analyses of dietary patterns and dietary adaptations in women with and without GDM from the Growing Up in New Zealand study.

The characteristics of women participating in the Growing Up in New Zealand study have been described previously [42]. The selection of participants included in this study is shown in Figure 1. Socio-demographic, health and lifestyle characteristics of the 5384 women included in the analyses of this study are shown in Table 1. GDM was identified in 280 (5.2%) of women. There were significant differences in maternal age, ethnicity, socioeconomic deprivation, pre-pregnancy BMI, physical activity pre-pregnancy and during the first trimester, pre-pregnancy dieting status, smoking patterns and alcohol consumption between women with and without GDM (Table 1).

Dietary pattern scores differed between women diagnosed with GDM and those without GDM (Table 2). Women with GDM had significantly lower mean scores for ‘Junk’ and ‘Traditional/White bread’ dietary patterns and a significantly higher mean score for the ‘Fusion Protein’ dietary pattern. Logistic regression analysis showed higher scores on the ‘Junk’ OR (per 1 SD change) 0.61 (95% CI; 0.51, 0.74) *p* ≤ 0.0005 and the ‘Traditional/White bread’ dietary patterns OR 0.89 (0.71, 0.93) *p* = 0.002 were associated with a decreased odds of having GDM and higher scores on the ‘Fusion/Protein’ dietary pattern OR 1.25 (1.13, 1.38) *p* ≤ 0.0005 were associated with an increased odds of having GDM. Although not statistically significant, there was a strong trend of a higher score on the ‘Health conscious’ dietary pattern to be associated with a reduced likelihood of having GDM OR 0.89 (0.78, 1.00) *p* = 0.055 in unadjusted analyses. After adjusting for maternal age, ethnicity, socioeconomic deprivation, pre-pregnancy BMI, pre-pregnancy and first trimester physical activity, smoking patterns, alcohol consumption and dietary pattern score on alternative dietary patterns, higher scores on ‘Junk’ aOR 0.64 (0.52, 0.80) *p* = 0.001 and ‘Traditional/White bread’ aOR 0.66 (0.55, 0.78) *p* ≤ 0.0005 dietary patterns remained significantly associated with a reduced likelihood of having GDM, while the relationship between scores on the ‘Fusion/Protein’ and ‘Health conscious’ dietary patterns were attenuated and not significantly associated with GDM status aOR 1.04 (0.90, 1.2) *p* = 0.269 and aOR 1.11 (0.96, 1.29) *p* = 0.378 respectively.

Comparing scores in the highest versus the lowest tertile for each dietary pattern showed similar results (Table 3). Women with dietary pattern scores in the highest tertiles of ‘Junk’ were 62% less likely, and ‘Traditional/White bread’ 40% less likely to have GDM compared to women in the lowest tertiles. Having a score in the highest tertile of the ‘Fusion Protein’ dietary pattern almost doubled the likelihood of having a GDM diagnosis in unadjusted analyses. After adjusting for potential confounders (maternal age, ethnicity, socioeconomic deprivation, pre-pregnancy BMI, pre-pregnancy and first trimester physical activity, smoking pattern, alcohol consumption and dietary pattern score on alternative dietary patterns), women with scores in the highest tertiles of the ‘Junk’ and ‘Traditional/White bread’ dietary patterns were half as likely to have a diagnosis of GDM compared to women with scores in the lowest tertiles. The higher likelihood of GDM for those with scores in the highest tertile of the ‘Fusion Protein’ dietary pattern compared to the lowest tertile was attenuated and no longer statistically significant in the adjusted model. The ‘Health conscious’ dietary pattern was not significantly associated with GDM in both the unadjusted and adjusted models when comparing women with scores in the highest versus the lowest tertiles; however, the relationship of a reduced likelihood of GDM in the unadjusted model was reversed to an increased likelihood of GDM in the adjusted model.

In analyses stratified according to the timing of GDM diagnosis, the relationship between higher scores on ‘Junk’ and ‘Traditional/White bread’ dietary patterns with a reduced likelihood of GDM strengthened when comparing women without GDM to women with GDM diagnosed before the antenatal interview (Table 4). In analyses comparing women diagnosed with GDM after the antenatal interview to women without GDM, only the association of a higher score on the ‘Junk’ dietary pattern with a reduced likelihood of GDM diagnosis remained significant, although this was attenuated.

Differences in the types of foods or drinks avoided or added due to pregnancy between women with and without GDM and between women diagnosed before or after the antenatal interview are shown in Table 5. Significantly more women with GDM avoided chocolate, foods high in fat or sugar and soft drinks and added milk or milk products to their diets during pregnancy compared to women without GDM. Significantly more women with GDM diagnosed before the antenatal interview avoided high fat or sugar foods and added milk or milk products compared to women with GDM diagnosed after the antenatal interview.

Almost three-quarters (71.6%) of women reported receiving information that resulted in making changes to their diet. Sources of information leading to dietary change in women with and without GDM are presented in Table 6. Significantly more women with GDM reported receiving information from a dietitian or nutritionist or an obstetrician and significantly fewer from friends, antenatal class, or books, magazines and newspapers compared to women without GDM. Compared to women without GDM, the magnitude of these differences was greater in women with GDM diagnosed before the antenatal interview than those with GDM diagnosed after the antenatal interview. Almost eight times more women with GDM diagnosed before the antenatal interview reported receiving information from a dietitian or nutritionist compared to women with GDM diagnosed after the antenatal interview.

## 4. Discussion

In this cohort of New Zealand women, we found women with a diagnosis of GDM had significantly lower adherence to ‘Junk’ and ‘Traditional/White bread’ dietary patterns compared to women without a diagnosis of GDM. These findings are in contrast to a number of studies exploring dietary patterns associated with GDM [26,28,29,30] in which dietary patterns characterized by higher intakes of processed meats, fried foods, cakes and biscuits, confectionary, jams, full-fat dairy and salty snacks, similar to the ‘Junk’ and ‘Traditional/White bread’ dietary patterns identified in the Growing Up in New Zealand cohort, have been associated with an increased risk of GDM. Together with our finding that a significantly greater proportion of women with GDM than of those without GDM reported receiving information from a dietitian or nutritionist or obstetrician and avoiding foods or drinks high in fat or sugar, these results are strongly suggestive of a treatment effect. This is particularly evident when looking at stratified analyses according to timing of diagnosis in which these relationships were strongest in women diagnosed with GDM before the antenatal interview compared to those diagnosed after, suggesting that these women with GDM are likely to have received advice on the management of GDM and made dietary adaptations prior to the completion of the food frequency questionnaire.

Diet is considered pivotal in the management of GDM [55,56,57] and New Zealand guidelines recommend women with a diagnosis of GDM are referred to a specialist diabetes in pregnancy service where they receive specialist care from a multidisciplinary team, including input from a dietitian and obstetrician [58]. Dietary guidelines for the management of GDM frequently recommend a low glycaemic index diet or the avoidance of simple sugars [59,60,61,62,63] and some encourage a reduction in saturated fats [58,59,63]. In our previous work exploring dietetic practice in the management of GDM, dietitians frequently reported discussing healthy eating, core food group requirements, carbohydrate quantity and distribution, simple sugars and fat with women with GDM [64]. These recommendations are consistent with the changes that women reported making to their diets and the differences seen in dietary patterns. For example, advice to limit intake of simple carbohydrates and saturated fats could result in lower scores on the ‘Junk’ and ‘Traditional/White bread’ dietary patterns as foods with these characteristics had high factor loadings in these patterns. Similarly, associations of higher intakes on the ‘Fusion/Protein’ dietary patterns in women with GDM diagnosed before the antenatal interview could be due to the foods with high factor loadings in this pattern being noodles, rice, or pasta (potentially low glycaemic index foods), seafood, chicken, red meat, eggs and green leafy vegetables, which women may have been encouraged to consume when receiving dietary advice for GDM. The ‘Health Conscious’ dietary pattern included high factor loadings for dried fruits and non-citrus fruits, which some women may limit after learning of their diagnosis of GDM due to the sugar content of these foods. This may partly explain non-significant and inconsistent findings for this dietary pattern. The smaller proportion of women avoiding soft drink in those with GDM diagnosed before the antenatal interview compared to those diagnosed after the antenatal interview could be explained by the finding that dietitians in New Zealand frequently provide advice on artificial sweeteners [64]. It is possible that women receiving this type of advice choose artificially sweetened soft drinks rather than avoiding them. Dietitians in our survey of dietetic practice also commonly reported discussing calcium and core food group requirements with women with GDM [64], consistent with a greater proportion of women with GDM reporting adding milk and milk products to their diets.

The relationship between higher adherence to the ‘Junk’ and ‘Traditional/White bread’ dietary patterns and a reduced likelihood of having a diagnosis of GDM, even in analyses including only women with GDM diagnosed after the interview and, therefore, presumably unaware of their diagnosis, may be explained by the finding that there was still a greater proportion of women diagnosed with GDM after the interview who reported receiving information from a dietitian or nutritionist compared to women without GDM. In our survey of dietetic practice in the management of GDM, 76% of dietitians reported that women had already received nutrition information prior to their first encounter with a dietitian [64]. Furthermore, a greater proportion of women with GDM reported a pre-pregnancy BMI in the overweight and obese category, receiving fertility treatment, including weight loss advice, and to be actively dieting pre-pregnancy compared to women without GDM. It is possible that women with GDM may have been identified or self-identified as having risk factors for GDM or other pregnancy complications and were therefore already actively making changes to their diets prior to receiving a diagnosis of GDM.

The diagnosis of GDM has been described as a ‘teachable moment’, in which a diagnosis of GDM may motivate women to make health-related behavioural changes [65]. These findings and ours are supported by studies in which nutrition counselling in pregnant women with or at risk of GDM have resulted in favourable dietary changes [66,67,68]. In a cluster-randomized controlled trial, Kinnunen et al. (2014) investigated the impact of intensified dietary counselling on food habits of 399 women at risk of GDM. The intervention consisted of five individual counselling sessions on gestational weight gain, physical activity and diet by public health nurses during routine visits to maternity clinics in Finland and resulted in improvements in consumption of fruit and vegetables, high fibre bread and low fat cheese and in the quality of dietary fat intake when compared to women in the usual care group. In a group of Canadian women, 17 with GDM and 27 with normal glucose tolerance, Morisset et al. (2014) demonstrated that a multidisciplinary medical and nutrition intervention, including counselling from a registered dietitian, was effective in the achieving prescribed macronutrient distributions and controlling gestational weight gain in women with GDM. Other studies have demonstrated further benefits of dietetic input in women with GDM, including reduced insulin use and improvements in glycated haemoglobin [69] and reduced likelihood of infant admission to neonatal intensive care or special care units [70].

Strengths of this study include the large, ethnically diverse sample size and the availability of information on factors likely to impact on dietary intake such as sources of information leading to dietary change during pregnancy. In the comparisons of women with GDM diagnosed before and those diagnosed after the antenatal interview, only women with a GDM diagnosis according to laboratory results were included in analyses, as clinical coding data did not include the exact date of diagnosis. A limitation to our findings is that data were not collected on actual input received during pregnancy to confirm our hypotheses. We also had insufficient data on history of GDM in a previous pregnancy to determine whether this may have contributed to dietary changes during the pregnancy reported on during this study.

## 5. Conclusions

Our study found women with GDM had significantly lower adherence scores on ‘Junk’ and ‘Traditional/White bread’ dietary patterns compared to women without GDM. A greater proportion of women with GDM avoided foods and drinks high in fat or sugar and reported receiving dietary information from a dietitian or nutritionist or an obstetrician compared to women without GDM. Women with GDM appear to make significant changes to their diet during pregnancy, most likely as a result of advice from dietitians or nutritionists and obstetricians.

## Figures and Tables

**Table 1:** Characteristics of women in the Growing Up in New Zealand cohort according to gestational diabetes mellitus (GDM) diagnosis ^1^.

	Women Without GDM	Women With GDM	*p*-Value
*n* (%)	5104 (94.8)	280 (5.2)	
Age group (years)			<0.0005
<20	259 (5.1)	<10 (1.8)	
20–24	747 (14.6)	28 (10.0)	
25–29	1267 (24.8)	51 (18.2)	
30–34	1616 (31.7)	95 (33.9)	
35–39	1039 (20.4)	81 (28.9)	
40 and over	176 (3.4)	20 (7.1)	
Self-prioritised ethnicity			<0.0005
European	2913 (57.2)	103 (36.8)	
Māori	686 (13.5)	22 (7.9)	
Pacific	630 (12.4)	55 (19.6)	
Asian	681 (13.4)	84 (30.0)	
Other	186 (3.6)	16 (5.7)	
Parity			0.628
First child	2167 (42.5)	123 (43.9)	
Subsequent child	2937 (57.5)	157 (56.1)	
Pregnancy planning			0.857
Planned	3144 (61.8)	171 (61.3)	
Unplanned	1941 (38.2)	108 (38.7)	
Fertility treatment			0.090
Yes	299 (9.5)	23 (13.5)	
No	2844 (90.5)	148 (86.5)	
Fertility treatment: fertility awareness and weight loss			0.169
Yes	26 (8.7)	<10 (17.4)	
No	272 (91.3)	19 (82.6)	
Socioeconomic deprivation			0.021
1 to 2 (least deprived)	864 (16.9)	30 (10.7)	
3 to 4	978 (19.2)	45 (16.1)	
5 to 6	909 (17.8)	61 (21.8)	
7 to 8	1050 (20.6)	63 (22.5)	
9 to 10 (most deprived)	1301 (25.5)	81 (28.9)	
Highest education			0.266
No secondary school	319 (6.3)	14 (5.0)	
Secondary school/NCEA* 1–4	1187 (23.3)	77 (27.6)	
Diploma/Trade certificate/NCEA* 5–6	1550 (30.4)	76 (27.2)	
Bachelor’s degree	1178 (23.1)	58 (20.8)	
Higher degree	861 (16.9)	54 (19.4)	
Pre-pregnancy BMI (kg/m^2^)			<0.0005
<18.5	192 (4.2)	<10 (2.8)	
18.5–24.9	2558 (56.2)	103 (41.4)	
25–29.9	1034 (22.7)	60 (24.1)	
30 and over	767 (16.9)	79 (31.7)	
Gestational weight gain			0.005
Gained ≥5 kg	4455 (88.9)	229 (83.0)	
Gained <5 kg	376 (7.5)	32 (11.6)	
No change	43 (0.9)	<10 (1.1)	
Lost <5 kg	74 (1.5)	10 (3.6)	
Lost ≥5 kg	62 (1.2)	<10 (0.7)	
Actively dieting pre-pregnancy	1272 (24.9)	104 (37.1)	<0.0005
Pre-pregnancy dieting weight loss			0.300
Yes	1032 (82.7)	81 (78.6)	
No	216 (17.3)	22 (21.4)	
Physical activity†			
Physically active pre-pregnancy	2578 (50.5)	119 (42.5)	0.009
Physically active during first trimester	1459 (28.6)	62 (22.1)	0.020
Physically active during second and third trimesters	1147 (22.5)	59 (21.1)	0.584
Smoking patterns			0.012
Continued smoking during pregnancy	508 (10.0)	13 (4.6)	
Stopped smoking during pregnancy	494 (9.7)	31 (11.1)	
Non-smoker	4088 (80.3)	236 (84.3)	
Alcohol consumption			<0.0005
Any drinking during pregnancy	1536 (30.1)	42 (15.0)	
Stopped drinking during pregnancy	2276 (44.6)	100 (35.7)	
Non-drinker	1287 (25.2)	138 (49.3)	

^1^ Includes only women interviewed before the birth of their child and excludes women with other forms of diabetes or for whom diabetes status could not be determined or those without dietary pattern scores; data presented as number of participants (percentages), missing values have not been included in the column%; *NCEA is the primary national qualification for secondary school students in New Zealand; †Engaged in moderate or vigorous physical activity for at least 150 min per week; BMI, Body Mass Index; NCEA, National Certificate of Educational Achievement.

**Table 2:** Dietary pattern scores among women with and without GDM.

Dietary Pattern	Women Without GDM *n* = 5104	Women With GDM *n* = 280	*p*-Value
Junk	0.02 (1.01)	−0.28 (0.95)	<0.0005
Health conscious	0.01 (1.00)	−0.11 (0.95)	0.055
Traditional/White bread	0.01 (1.01)	−0.18 (0.93)	0.002
Fusion Protein	−0.02 (0.99)	0.26 (1.09)	<0.0005

Data presented as the mean (SD).

**Table 3:** Unadjusted and adjusted odds of having GDM for women with intakes in the highest tertile compared to those with intakes in the lowest tertile of each dietary pattern.

Dietary Pattern	*n*	OR (95% CI)	*p*-Value	*n*	aOR (CI)	*p*-Value
Junk	3581	0.38 (0.28, 0.52)	<0.0005	3154	0.49 (0.34, 0.70)	<0.0005
Health conscious	3580	0.80 (0.60, 1.08)	0.141	3134	1.24 (0.87, 1.77)	0.244
Traditional/White bread	3597	0.60 (0.44, 0.81)	0.001	3157	0.47 (0.32, 0.68)	<0.0005
Fusion Protein	3589	1.93 (1.42, 2.62)	<0.0005	3160	1.25 (0.87, 1.81)	0.231

OR (95% CI) from unadjusted logistic regression; aOR (95% CI) from adjusted logistic regression (maternal age group, ethnicity, socioeconomic deprivation, pre-pregnancy BMI, pre-pregnancy and first trimester physical activity, smoking, alcohol consumption and dietary patterns included in the model).

**Table 4:** Adjusted odds of having GDM for women with intakes in the highest tertile compared to those with intakes in the lowest tertile of each dietary pattern stratified according to timing of diagnosis.

	GDM Diagnosed Before Interview			GDM Diagnosed After Interview		
Dietary Pattern	*n*	aOR (CI)	*p*-Value	*n*	aOR (CI)	*p*-Value
Junk	3050	0.27 (0.15, 0.50)	<0.0005	3037	0.54 (0.30, 0.96)	0.036
Health conscious	3043	0.95 (0.54, 1.68)	0.860	3035	1.44 (0.81, 2.58)	0.214
Traditional/White bread	3072	0.21 (0.12, 0.38)	<0.0005	3045	0.64 (0.35, 1.18)	0.153
Fusion Protein	3062	1.13 (0.64, 1.99)	0.676	3055	0.67 (0.38, 1.18)	0.169

aOR (95% CI) from adjusted logistic regression (maternal age group, ethnicity, socioeconomic deprivation, pre-pregnancy BMI, pre-pregnancy and 1st trimester physical activity, smoking, alcohol consumption and dietary patterns included in the model).

**Table 5:** Foods and drinks avoided or added during pregnancy.

	Women Without GDM	Women With GDM	GDM Diagnosed before Interview	GDM Diagnosed after Interview
Foods/drinks avoided	*n* = 4456	*n* = 241	*n* = 104	*n* = 96
Chocolate	29 (0.7)	<10 (3.7) ^a^	<10 (4.8) ^a^	<10 (3.1) ^a^
High fat or sugar foods	252 (5.7)	61 (25.3) ^a^	37 (35.6) ^a,b^	11 (11.5) ^a,b^
Alcohol	2876 (64.5)	109 (45.2) ^a^	52 (50.0) ^a^	42 (43.8) ^a^
Soft drinks	742 (16.7)	78 (32.4) ^a^	30 (28.8) ^a^	33 (34.4) ^a^
Foods/drinks added	*n* = 2129	*n* = 116	*n* = 50	*n* = 48
Vegetables and fruit	108 (5.1)	<10 (1.7)	<10 (0.0)	<10 (2.1)
Breads and cereals	126 (5.9)	<10 (7.8)	<10 (10.0)	<10 (6.3)
Milk or milk products	779 (36.6)	62 (53.4) ^a^	32 (64.0) ^a,b^	21 (43.8) ^b^
Lean meat, chicken, seafood, eggs, cooked dried beans or peas	766 (36.0)	45 (38.8)	18 (36.0)	23 (47.9)
Fluids	831 (39.0)	28 (24.1) ^a^	10 (20.0) ^a^	13 (27.1)
Supplements	446 (20.9)	24 (20.7)	<10 (12.0)	12 (25.0)
Other	337 (15.8)	13 (11.2)	<10 (12.0)	<10 (8.3)

Data presented as number of participants (percentages), missing have not been included in the column%; ^a^ Significantly different in women with GDM compared to women without GDM (*p* < 0.05); ^b^ Significantly different between women with GDM diagnosed before vs. after the antenatal interview (*p* < 0.05).

**Table 6:** Information leading to dietary changes.

	Women Without GDM	Women With GDM	Women With GDM Diagnosed before Interview	Women With GDM Diagnosed after Interview
*n*	5100	280	124	109
Received information leading to dietary changes	3652 (71.6)	215 (76.8)	98 (79.0)	83 (76.1)
Sources of information				
Family/whānau	873 (23.9)	36 (16.7)	14 (14.3) ^a^	16 (19.3)
Friends	853 (23.4)	30 (14.0) ^a^	15 (15.3)	11 (13.3) ^a^
GP	1274 (34.9)	71 (33.0)	31 (31.6)	29 (34.9)
Midwife	2703 (74.1)	151 (70.2)	62 (63.3) ^a^	63 (75.9)
Obstetrician	273 (7.5)	27 (12.6) ^a^	16 (16.3) ^a^	<10 (10.8)
Dietitian or nutritionist	61 (1.7)	58 (27.0) ^a^	46 (46.9) ^a,b^	<10 (6.0) ^a,b^
Alternative health practitioner	58 (1.6)	<10 (1.9)	<10 (1.0)	<10 (3.6)
Antenatal class	247 (6.8)	<10 (2.8) ^a^	<10 (1.0) ^a^	<10 (4.8)
The internet	681 (18.7)	33 (15.3)	13 (13.3)	17 (20.5)
Radio	26 (0.7)	<10 (0.5)	<10 (0.0)	<10 (1.2)
TV	123 (3.4)	<10 (1.4)	<10 (2.0)	<10 (1.2)
Books, magazines, newspaper	1117 (30.6)	48 (22.3) ^a^	18 (18.4) ^a^	25 (30.1)
Other	127 (3.5)	10 (4.7)	<10 (7.1) ^b^	<10 (0.0) ^b^

Data presented as number of participants (percentages), missing have not been included in the column%; ^a^ Significantly different between women with GDM compared to women without GDM (*p* < 0.05); ^b^ Significantly different between women with GDM diagnosed before versus after the antenatal interview (*p* < 0.05).
